# Prevalence and molecular characterization of *Mycobacterium tuberculosis* complex in cattle and humans, Maiduguri, Borno state, Nigeria: a cross-sectional study

**DOI:** 10.1186/s12866-022-02710-y

**Published:** 2023-01-09

**Authors:** Ayi Vandi Kwaghe, James Agbo Ameh, Caleb Ayuba Kudi, Abdul-Ganiyu Ambali, Hezekiah Kehinde Adesokan, Victor Oluwatoyin Akinseye, Olubukola Deborah Adelakun, Joy Gararawa Usman, Simeon Idowu Cadmus

**Affiliations:** 1 Department of Veterinary and Pest Control Services, Federal Ministry of Agriculture and Rural Development, P. M. B. 135, Area 11, Garki, Abuja, Nigeria; 2 Nigeria Field Epidemiology and Laboratory Training Programme, Abuja, Nigeria; 3 Department of Veterinary Microbiology, Faculty of Veterinary Medicine, University of Abuja, Abuja, Nigeria; 4 Department of Public Health and Preventive Medicine, Faculty of Veterinary Medicine, Ahmadu Bello University Zaria, Zaria, Kaduna State Nigeria; 5 Department of Veterinary Medicine, Faculty of Veterinary Medicine, University of Ilorin, Ilorin, Kwara State Nigeria; 6 Department of Public Health and Preventive Medicine, Faculty of Veterinary Medicine, University of Ibadan, Ibadan, Oyo State Nigeria; 7 Department of Chemical Sciences, Augustine University Ilara-Epe, Epe, Lagos State Nigeria; 8 National Veterinary Research Institute, Vom, Plateau State Nigeria

**Keywords:** Prevalence, Bovine and human tuberculosis, Culture and isolation, Genus typing, Deletion analysis, Spoligotyping, *Mycobacterium tuberculosis* complex

## Abstract

**Introduction:**

Globally, the highest burden of bovine and human tuberculosis resides in Africa and Asia. Tuberculosis (TB) is the second leading single infectious killer after severe acute respiratory syndrome corona virus-2 (SARSCOV-2). Bovine TB remains a treat to wild and domesticated animals, humans and hinders international trade in endemic countries like Nigeria. We aimed at determining the prevalence of bovine and human tuberculosis, and the spoligotypes of *Mycobacterium tuberculosis* complex in cattle and humans in Maiduguri.

**Methods:**

We conducted a cross sectional study on bovine and human tuberculosis in Maiduguri, Borno state. We calculated sample size using the method of Thrusfield. Lesions suggestive of TB from 160 slaughtered cattle were obtained from Maiduguri Central Abattoir. Sputum samples from humans; 82 abattoir workers and 147 suspected TB patients from hospitals/clinics were obtained. Lesions and sputum samples were cultured for the isolation of *Mycobacterium* spp. Positive cultures were subjected genus typing, deletion analysis and selected isolates were spoligotyped. Data was analysed using SPSS VERSION 16.0.

**Results:**

Prevalence of 32.5% (52/160) was obtained in cattle. Damboa local government area (LGA), where majority of the infected animals were obtained from had 35.5% bTB prevalence. All categories analysed (breed, age, sex, body conformation and score) had *P*-values that were not significant (*P* > 0.05). Sputum culture revealed a prevalence of 3.7% (3/82) from abattoir workers and 12.2% from hospitals/clinics. A significant *P*-value (0.03) was obtained when positive culture from abattoir and that of hospitals/clinics were compared. Out of the 52 culture positive isolates obtained from cattle, 26 (50%) belonged to *M. tuberculosis* complex (MTC) and 17/26 (65.4%) were characterized as *M. bovis*. In humans, 7/12 (58.3%) MTC obtained were characterized as *M. tuberculosis*. Spoligotyping revealed SB0944 and SB1025 in cattle, while SIT838, SIT61 of LAM10_CAM and SIT1054, SIT46 of Haarlem (H) families were obtained from humans.

**Conclusions:**

Cattle in Damboa LGA need to be screened for bTB as majority of the infected animals were brought from there. Our findings revealed the presence of SB0944 and SB1025 spoligotypes from cattle in Borno state. We isolated *M. tuberculosis* strain of the H family mainly domiciled in Europe from humans.

**Supplementary Information:**

The online version contains supplementary material available at 10.1186/s12866-022-02710-y.

## Background

Globally, in the animal sector, the highest prevalence of bovine tuberculosis (bTB) is in Africa and parts of Asia while the disease is also found in countries in Europe and the Americas [1]. The World Organisation for Animal Health through the World Animal Health Information System (WAHIS) from January 2017 to June 2018, received reports on bTB from 44% of countries associated with OIE and only a quarter of the affected countries applied relevant control measures [1]. Bovine tuberculosis is a chronic disease of cattle caused by *Mycobacterium bovis*. The disease has significant impact on the international cattle trade as well as public health [2]. *Mycobacterium bovis* can also infect and cause disease in many other mammals; humans, deer, goats, pigs, cats, dogs, and wildlife species such as wild boars, deer, and antelopes. Tuberculosis caused by *M. bovis* is not clinically distinguishable from TB due to *M. tuberculosis* [3, 4].

In humans, tuberculosis (TB) is the 13th leading cause of death and 2nd leading infectious killer after corona virus-2019 (COVID-19) [5, 6]. One quarter of the global population is infected with *Mycobacterium tuberculosis* [6]. Globally, in 2020, 10 million people were infected with TB and 1.5 million died of the disease [5] and 86% of new TB cases were from the 30 high TB burden countries with eight countries accounting for 2/3rd of the new cases; India, China, Indonesia, the Philippines, Pakistan, Nigeria, Bangladesh and South Africa [5]. There is a global decline of TB incidence with cumulative reduction rate of 11% about half of the End TB strategy of the stipulated 20% reduction from 2015 to 2020 [5]. Ending the TB epidemic by 2030 is one of the health targets of the United Nations Sustainable Development Goals (SDGs) [5].

Tuberculosis disproportionately affects people in resource-poor settings especially in Africa and Asia, posing significant challenges to developing economies as it primarily affects people during their most productive years with more than 90% of new TB cases and deaths occurring in developing countries [7]. Nigeria ranked 6th amongst the 30 high TB burden countries globally, and first in Africa [8]. Nigeria is also among the 14 countries that are in all the three WHO Global high-burden country lists for TB, TB/human immune deficiency virus (HIV) and multi-drug resistant tuberculosis (MDR-TB) with an estimated incident rate of 219 per 100,000 population and mortality rate of 64/100,000 excluding people living with HIV. Nigeria accounts for 4% of the total TB global burden [8].

In developing countries, laboratory diagnosis of TB is often limited to the smear microscopy in humans, thus limiting the estimation of the role of *M. bovis* in human infection [9, 10]. Introduction of DNA fingerprinting techniques for *M. tuberculosis* has largely enhanced the understanding of TB transmission [11]. Differentiation of members of *M. tuberculosis* complex (MTC) is relevant for accurate diagnosis of mycobacterial diseases, public health surveillance and effective case management [12]. Differentiation of MTC has become particularly important in adult and pediatric patients with human immunodeficiency virus (HIV)-related immune suppression [12] and occupationally exposed individuals such as abattoir workers. People living with HIV and infected with TB due to *M. bovis* are twice more likely to die during treatment than those infected with *M. tuberculosis* [12]*.*

Tuberculosis and other mycobacterial infections are major opportunistic infections in HIV/AIDS infected individuals while HIV/AIDS is a major predisposing factor for TB including reactivations of the disease [13, 14]. Even though the risk to human health is low in most developed countries, the HIV pandemic raises concern about its impact on the transmission of *M. bovis* to and between humans [15]. The highest risk groups are individuals with concomitant HIV/AIDS infection [16]. Cases of HIV-related human TB due to *M. bovis* have been reported in many developed countries [17, 18]. Limited data is available regarding the spread of bTB amongst human population in developing countries; global estimates of 2.1% of pulmonary TB and 9.4% of extra-pulmonary TB cases is attributed to *M. bovis*. Estimates of some studies in Africa attributes *M. bovis* infection to about 5–7% of all human TB cases in the region [19, 20]. Studies conducted over the past 30 years in Nigeria revealed prevalence ranges from 2.5% in 1976 to 14% in 2007 for *M. bovis*, indicating increase in the prevalence of bTB over the years [21]. Molecular analysis of mycobacterial strains isolated from both pulmonary and extra pulmonary TB cases have indicated that up to 14% of them belong to *M. bovis* [21].

Some diagnostic tools used in the speciation of MTC are deletion typing and spoligotyping. Deletion typing is a multiplex PCR technique that differentiates members of the MTC by the amplification of genomic regions of difference (RD1, RD4, RD9, and RD12) thereby identifying specific strains based on the presence and/or absence of RD-region [22]. Spoligotyping is a very practical and reproducible PCR-based method, which assays the presence or the absence of a set of target sequences in the direct repeat (DR) locus [23]. This technique is based on the amplification of the DR region and subsequent differential hybridization of the amplified products with membrane-bound oligonucleotides complementary to the variable spacer regions localized between the DR’s [24]. Strains that are similar or different can be distinguished by their spoligotype patterns which are characterized by the number and identity of spacers [24]. The presence of the spacer sequences varies in different strains and are visualized by a spot on a fixed site of the hybridization membrane [23]. In Nigeria, only few studies [25–29] have so far been conducted to classify MTC despite the prevalent risk of inter-transmission between cattle and livestock workers in the country. Maiduguri is known to domicile many of the cattle slaughtered in Nigeria as supplies of these animals are made to various parts of the country. It is also characterized by high livestock activities including slaughter and processing with concomitant human-livestock interactions. Despite the prevalence of bTB, 10.7% in cattle [30] and 0.2% in humans [31]; circulating MTC strains among cattle and humans in Maiduguri are largely unknown. Our hypothesis was; the spoligotypes of MTC circulating in cattle and humans in Maiduguri is the same with the spoligotypes circulating in other parts of the country. The study aimed at characterizing MTC isolates from cattle and humans in Maiduguri, to provide important insights into the epidemiology of bTB in the area. We aimed at determining the prevalence of bovine and human tuberculosis and the spoligotypes of *Mycobacterium tuberculosis* complex in cattle and humans, in Maiduguri.

## Materials and methods

### The design and setting of the study

**Fig. 1:**
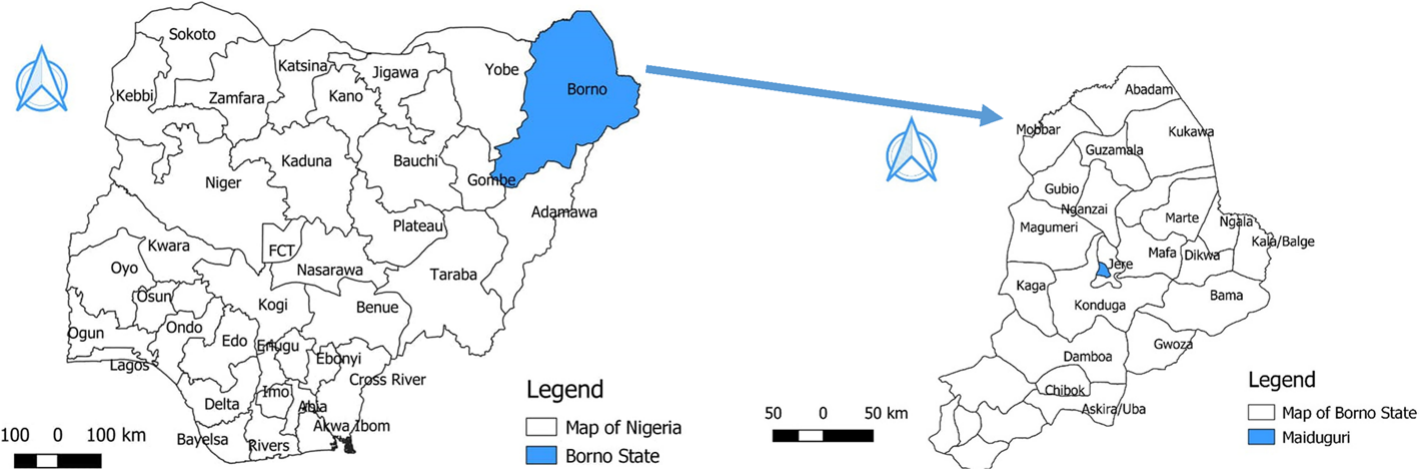
Map of Nigeria indicating Borno State (blue) and Map of Borno State showing Maiduguri (blue), the study area

Maiduguri, the capital of Borno State, located in the North East region of Nigeria (Fig. 1). It is the largest city in Borno State having a population of about 1,112,449 inhabitants [32]. The state shares international borders with Cameroon, Chad and Niger Republic. The major abattoir in the state is located in Maiduguri where an average of 200 cattle is slaughtered daily. The Maiduguri abattoir is the only abattoir in the metropolitan and suitable for the study because cattle from all parts of the state and across international borders (Chad republic, Niger and Cameroon) are brought to the abattoir. Data from the National Tuberculosis and Leprosy Training Programme indicates that Borno State has 252 Directly Observed Therapy Shortcourse (DOTS) Centres out of which 54 are domiciled in Maiduguri. Geographical Positioning System (GPS, GARMIN‘s eTrex Legend personal navigator) was used in determining the location of the sampled sites; Maiduguri Abattoir (longitude 13.17859°E and latitude 11.858611^O^N). Other study sites where sputum samples were collected include; the Chest Clinic, Sir Kashim Ibrahim Road Maiduguri (longitude 13.14565°E and latitude 11.83814^O^N); Chest Hospital Ruwan Zafi, Maiduguri (longitude 13.20222°E and latitude 11.85592^O^N); State Specialist Hospital Maiduguri (longitude 13.15013°E and latitude 11.83939^O^N); and the University of Maiduguri Teaching Hospital (UMTH) which is located along Bama road, Costin (Longitude 13.17898°E and Latitude 11.82606^O^N).

**Fig. 2:**
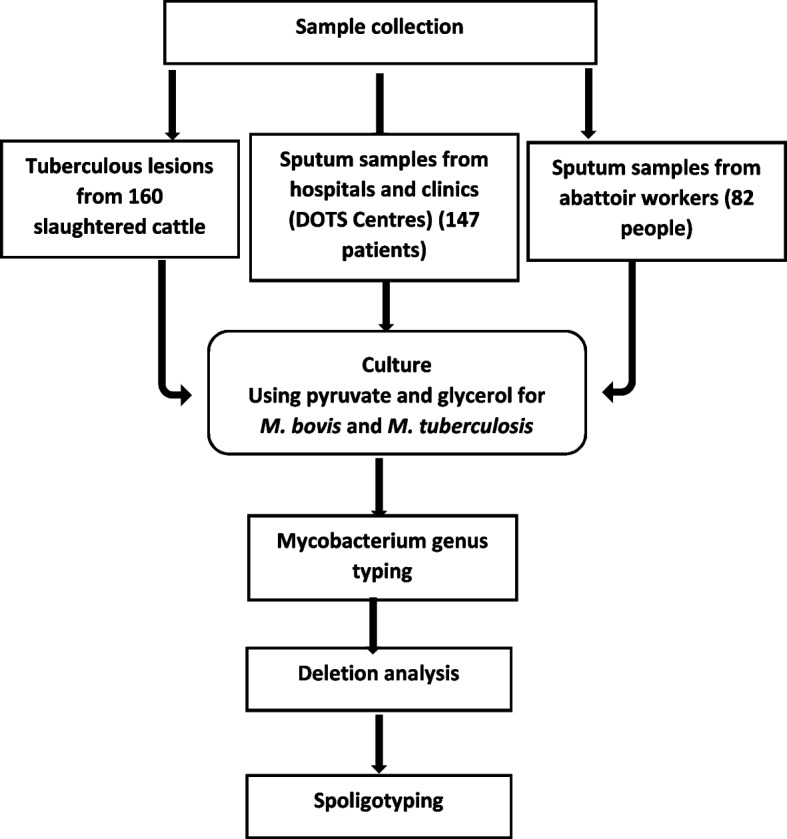
Diagrammatic depiction of procedures and analysis conducted

We conducted a cross-sectional study of bovine and human tuberculosis (Fig. 2). Inclusion criteria for the study were all cattle older than a year, taken to the abattoir for slaughter and indicated tuberculous-like lesions at postmortem while our exclusion criteria were cattle below a year that were brought to the abattoir for slaughter. For the abattoir workers, our inclusion criteria were all abattoir workers; staff, butchers, meat sellers at the abattoir that were willing and agreed to participate in the study. At the TB DOTS centres in the hospitals/clinics, our inclusion criteria were patients suspected to be infected with TB and were referred to the laboratory to submit their sputum sample for analysis and consented to be part of the study. Suspect case of the study were those cases that met the national TB case definitions; “a suspected pulmonary TB case is defined as any person coughing for 2 weeks or more, with or without symptoms of weight loss, tiredness, fever, night sweats, chest pain, shortness of breath, loss of appetite and coughing up blood while a suspected extra-pulmonary TB case is a person with symptoms depending on the affected organ, vertebral spine (back pain, swelling on spine); bone (long standing pain and swelling of the bone); Joints (painful joint swelling, usually affecting one joint); kidney and urinary tract (painful urination, blood in urine, frequent urination, lower back pain/loin pain); upper respiratory tract (hoarseness of voice, pain on swallowing); pleural membrane of lungs (chest pain, difficulty in breathing, fever); meninges of the brain (headache, persistent fever, neck stiffness, vomiting, irritability, convulsions, loss of consciousness); lymph node (painless swelling of the node, may drain pus)” [33]. The entire period of the study was from June 2013 to September, 2015. Research assistants were trained on sample collection of tuberculous lesions from tissues and organs of infected cattle while those that were engaged in human sputum collection were trained on sputum sample collection, to ensure that the samples were collected properly [34].

#### Sample collection, transportation and storage

The sample size used for this study was calculated according to the method described by Thrusfield [*n* = 1.96^2^ P_exp_ (1-P_exp_) / d^2^; where *n* = required sample size, P_exp_ = expected prevalence and d = the desired absolute precision (5%)] [35] based on previously reported prevalence of TB in cattle [9, 36, 37], abattoir workers [27] and hospital-based studies [38, 39]. Samples were obtained from slaughtered cattle that had tuberculous lesions in the lungs and lymphnodes and in the case of generalized TB from other infected organs such as the, liver, spleen, kidney, heart, intestine at the Maiduguri Abattoir; lesions were obtained according to the predilection sites as observed grossly during postmortem examination. Samples were purposively collected following detailed meat inspection over 3 months period. During this period, sensitization campaigns were conducted among abattoir workers to encourage them to participate in the study. Following due verbal consent obtained from prospective participants, sputum samples were aseptically collected using properly labelled sterile plastic specimen containers with top screw caps. From designated hospitals and clinics for the study, three sputum samples were collected per patient (one spot sample, one-morning sample and another spot sample, which were pooled together in clean sterile well-labelled plastic containers with cock screw caps. All sputum samples collected from the various study sites were stored at the University of Maiduguri Teaching Hospital. Finally, the samples were packaged with ice packs in Coleman transport boxes for effective transportation and transported to the Tuberculosis and Brucellosis Laboratories of the Department of Veterinary Public Health and Preventive Medicine, University of Ibadan, for processing.

#### Processing of samples

Cattle lesions and human sputum samples were decontaminated according to earlier described procedures [40]. Samples were processed using the BD BBL™ Mycoprep™ N-Acetyl L-Cysteine-sodium hydroxide (NALC-NaOH) decontamination method (BD BBL Mycoprep, 2000) to decontaminate and concentrate the samples to get deposits.

#### Preparation of the buffer and NALC reagent

The BBL™ Mycoprep™ phosphate buffer was prepared by pouring one packet of the buffer powder into a 500 ml volumentric flask. The flask was filled to the 500 ml line with sterile distilled water. The solution was transfered to a screw capped container and with the cap loosened, it was autoclaved at 121 °C for 15 minutes, cooled at room temperature and the cap tightened. The screw-cap on the Mycoprep Reagent bottle was loosened and the ample containing NALC was located, excess air from the bottle was released and the cap tightened. With the bottle held in an upright position, the bottle was squeezed until the ample broke. The bottle was shaken gently to dissolve the NALC, exessive agitation was avoided.

#### Decontamination test procedure

In a biological safety cabinet, equal volume of activated NALC was added to sputum sample in aerosol-free 15 ml centrifuge tube with screw cap, the cetrifuge tube was capped and rocked gently until the specimen liquefied. If the sample remained viscous, more NALC reagent was added and the mixing repeated. The mixture was allowed to stand at room temperature for 15 minutes with occassional gentle shaking. Buffer of three times (3x) equivalent of the sample was added to the mixture, mixed by rocking gently and centrifuged, for 15 minutes at 3000×g. All the supernatant were carefully decanted, small quantity of phosphate buffer was added and the sediment resuspended. The suspension was used for smear preparation and perfomance of mycobacteriological procedures.

The processing of lesions from tissue samples for culture was based on the OIE recommendation for digestion and decontamination procedures. The tissues were first homogenized by using a pestle and mortar as described by OIE [41], followed by decontamination in a 15 ml centrifuge tube containing equal amount of homogenized specimen and NALC (N- acetyl- L–cysteine) NaOH (containing 4% NaOH, 2.9% sodium citrate). The tube containing the mixture was allowed to stand for 15 minutes at room temperature until the specimen was digested followed by neutralization using 6 ml phosphate buffer. The mixture was then centrifuged at 3000×g for 15 minutes. The supernatant was carefully decanted; 2 ml of phosphate buffer was added to resuspend the sediment. Smears were made on the prepared culture media and incubated for culture.

#### Isolation of mycobacteria

**Fig. 3:**
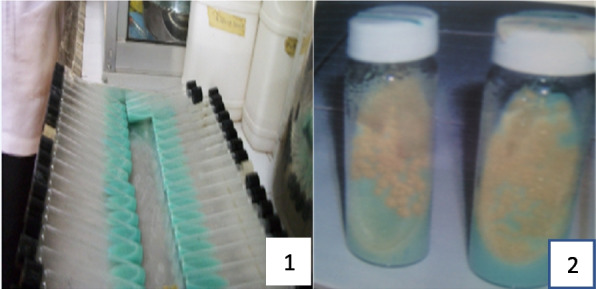
Prepared L-J media before the heating of the media (1) and L-J media with growth (2)

Culture and isolation of *Mycobacterium* species was conducted using Lowenstein Jensen media (L-J medium), an egg-based medium, with and without pyruvate (Fig. 3) [42]. The media were prepared in duplicate – one containing 0.5% pyruvate for the isolation of *M. bovis* and the other containing glycerol for the isolation of *M. tuberculosis.* The egg medium was prepared by dissolving 37.2 g of the L-J medium base powder in 600 ml of sterile distilled water, autoclaved and allowed to cool to about 45 °C - 60 °C. Homogenised eggs based on the quantity of L-J media to be prepared were thoroughly washed in water and then cleansed with methylated spirit before breaking and was aseptically added, thoroughly mixed and distributed in 10–15 ml volumes in sterile MacCartney bottles and the caps were securely fastened. The medium was inspissated in a slanted position to coagulate the already sterile medium (Fig. 3.1).

The two media types were properly labelled, dated and inoculated in duplicate (one on pyruvate containing medium and the other on glycerol containing medium) with the final sputum/tissue sediments spread evenly on the surfaces of pairs of slopes of L-J medium using Pasteur pipettes (two drops). All innoculated slants were incubated first in a slanting position for 24 hours to allow for even distribution of inoculum at 37 °C. The bottles were then re-arranged in an upright position to increase incubator space. Bottles were arranged in chronological order to make for easy examination. The bottles were incubated for 8 to 12 weeks for growth at 37 °C.

#### Examination of mycobacterial culture slants

All cultures were examined daily for the first 7 days after incubation to detect rapidly growing mycobacteria (non tuberculous mycobacteria) and also to detect contamination. Thereafter, the cultures were examined once a week for 8 to 12 weeks to detect positive cultures of mycobacteria before adjudging the culture to be negative if there was no growth. Cultures with completeley contaminated surfaces, liquified or discoloured were removed from the incubator, sterilised, discarded and excluded from the study.

Colonies from all resultant growths (Fig. 3.2) were examined for morphological appearance and acid-fast properties. Cultures with acid-fast bacilli properties were harvested into broth of 7H9 Middlebrook medium in a microcentrifuge tube and stored at -20 °C until needed for further investigation. The bacterial suspension of each isolate was stained by the Z-N technique as described ealier to confirm the presence of acid-fast bacilli in the resultant growth.

#### Microscopy (Ziehl-Neelsen staining method)

Loopful smears of final deposits were made on clean, grease-free, duly labelled dried slides. The smear was approximately 20 mm by 10 mm, corresponding to about 100 oil immerssion fields. Care was taken to ensure that the smears were not thick. The smears were left to dry naturally in the air before fixing over bunsen flame. The slides were stained by the ZN technique based on the ability of mycobacteria to retain basic dye when treated with mineral acid or an acid-alcohol solution [43]. The slides with fixed smears were arranged on staining rack over a sink. Freshly prepared carbol fuchsin was poured over the slides so that the smears were completely covered. The slides were then gently heated from below with a bunsen burner flame until steam rose and allowed to stay for about 5 minutes. The stained slides were washed with water under running tap and excess water on the slide was drained by tilting the slides. The slides were then replaced on the rack and the decolorizer (acid-alcohol) was poured over the slide to cover the smears and allowed to act for about 3 minutes. The slides were washed under the running tap. The counter-stain (methylene-blue) was poured on the slides and left to stay for about 1 minute before washing with water under running tap. The slides were drained, arranged vertically on a slide rack and allowed to dry naturally. Dried slides were examined under the oil-immerssion objective of a binocular microscope for the presence of acid fast bacilli, which appeared brick red against a blue background. The presence of AFB and cording of bacilli were indicative of *Mycobacterium* species. Positive cultures with acid-fast bacilli were harvested into a broth of 7H9 Middlebrook medium in microcentrifuge tubes and stored at -20 °C until further molecular analyses.

Mycobacterial isolates from culture-positive samples were heat killed at 80^C^C for 1 hour and used directly as DNA template for PCR, primers used are shown in Table 1. DNA amplification was done in a Thermocycler with each reaction mixture containing 2 μl DNA template, 5 μl of Q-buffer, 10X Buffer, 25 mM MgCl_2_, 4 μl × 10 mM dNTPs, 0.5 μl of each primer (50 pmol/ μl), 0.2 μl HotStarTaq DNA polymerase (Qiagen, Hilden, Germany) was made up to 25 μl with ultra-pure water. The reaction mixture was then heated in a Programme Thermal Controller (MyGene Series Peltier, Model MG 96^+^) using the following amplification programs: 95 °C for 15 min for enzyme activation, followed by 45 cycles at 94 °C for 1 min for denaturation, 62 °C for 1 min for annealing, and 72 °C for 1 min for the extension. After the last cycle, the samples were incubated at 72 °C for 10 min (Table 1). Thereafter, PCR amplification products were electrophoretically separated (fractionated) in 3.0% agarose in 1Xtbe pH 8·3 at 6 V/cm for 4 hours. A 1.5% agarose gel was prepared and the products were electrophoresed in 10 × TAE running buffer. Ethidium bromide at a ratio of 1:5, 100 bp DNA ladder, and orange 6x loading dye were used in gel electrophoresis. Finally, bands were visualized on a UV light cabinet. The primers MYCGEN-F and MYCGEN-R generate 1030 bp for genus detection common to MTBC members were MYCAV-R and MYCGEN-F generate 180 bp for the detection of *M. avium.* MYCINT-F and MYCGEN-R generate 850 bp for detection of *M. intracellulare*. TB1-F and TB1-R generate 372 bp for *M. bovis* detection [44], Table 1.

**Table 1:** List of oligonucleotide sequences and cycling conditions used in the study

Primer name	Primer sequence (oligonucleotide sequences)	Product size	Enzyme activation	Cycles [45]			Incubation
				Denaturation	Annealing	Extension	
MYCGEN-F MYCGEN-R	AGAGGTTGATCCTGGCTCAG TGCACACAGGCCACAAGGGA	1030 bp	95 °C 15 min.	94 °C 1 min	62 °C 1 min	72 °C 1 min	72 °C 10 min.
MYCGEN-F MYCAV-R	AGAGGTTGATCCTGGCTCAG ACCAGAAGACATGCGTCTTG	180 bp	95 °C 15 min.	94 °C 1 min	62 °C 1 min	72 °C 1 min	72 °C 10 min
MYCINT-F MYCGEN-R	CCTTTAGGCGCATGTCTTTA TGCACACAGGCCACAAGGGA	850 bp	95 °C 15 min.	94 °C 1 min	62 °C 1 min	72 °C 1 min	72 °C 10 min.
TB1-F TB1-R	GAACAATCCGGAGTTGACAA AGCACGCTGTCAATCATGTA	372 bp	95 °C 15 min.	94 °C 1 min	62 °C 1 min	72 °C 1 min	72 °C 10 min.

### Region of difference (RD) deletion typing

The PCR amplification procedures were carried out as earlier described [22]. All primers used are indicated in Table 2. Each reaction mixture consisted of 1 μl DNA template, 5 μl Q-buffer, 2.5 μl × 10 buffer, 2 μl 25 mM MgCl_2_, 4 μl × 10 mM dNTPs, 0.5 μl of each primer (50 pmol/ μl), 0.125 μl HotStarTaq plus DNA polymerase (Qiagen, Hilden, Germany) and was made up to 25 μl with ultra-pure water. The reaction mixture was then heated in a Programme Thermal Controller (MyGene Series Peltier, Model MG 96^+^) using the following amplification procedures: 95 °C for 15 min for enzyme activation, followed by 45 cycles at 94 °C for 1 min, 62 °C for 1 min, and 72 °C for 1 min. After the last cycle, the samples were incubated at 72 °C for 10 min (Table 2). PCR amplification products were electrophoretically fractionated in 3.0% agarose in 1Xtbe pH 8·3 at 6 V/cm for 4 h, and visualized by staining with ethidium bromide. Specific strains were identified based on the presence and/or absence of the genomic regions of difference (RD1, RD4, RD9, and RD12).

**Table 2:** List of oligonucleotide sequences and cycling conditions used in the study

Primer name	Primer sequence (oligonucleotide sequences)	Product size	enzyme activation	Cycles [45]			Incubation
				Denaturation	Annealing	Extension	
RD1	AAGCGGTTGCCGCCGACCGACC CTGGCTATATTCCTGGGCCCGG GAGGCGATCTGGCGGTTTGGGG	Present (146 bp)	95 °C 15 min.	94 °C 1 min	62 °C 1 min	72 °C 1 min	72 °C 10 min.
RD4	ATGTGCGAGCTGAGCGATG TGTACTATGCTGACCATGCG AAAGGAGCACCATCGTCCAC	Present (172 bp) Absent (268 bp)	95 °C 15 min.	94 °C 1 min	62 °C 1 min	72 °C 1 min	72 °C 10 min
RD9	CAAGTTGCCGTTTCGAGCC CAATGTTTGTTGCGCTGC GCTACCCTCGACCAAGTGTT	Present (235 bp) Absent (108 bp)	95 °C 15 min.	94 °C 1 min	62 °C 1 min	72 °C 1 min	72 °C 10 min.
RD12	GGGAGCCCAGCATTTACCTC GTGTTGCGGGAATTACTCGG AGCAGGAGCGGTTGGATATTC	Present (369 bp) Absent (306 bp)	95 °C 15 min.	94 °C 1 min	62 °C 1 min	72 °C 1 min	72 °C 10 min.

### Spoligotyping

Spoligotyping was done at the Division of Molecular Biology and Human Genetics, Stellenbosch University, South Africa. Spoligotyping was performed on isolates identified by deletion typing as members of MTC as previously described with minor modifications [23] (Tables 3 and 4). The direct repeat (DR) region was amplified by PCR with oligonucleotide primers derived from the DR sequence (Table 3). Then, 25 μl of the following reaction was used for the PCR: 12.5 μl of HotStarTag Master Mix (QIAGEN; this solution provided a final concentration of 1.5 mM MgCl_2_ and 200 μm each of deoxynucleoside triphosphate), 2 μl of each primer (20 pmol each), 5 μl of the suspension of heat-killed cells (approximately 10 to 50 ng) and 3.5 μl of distilled water. The mixture was heated for 15 minutes at 96 °C and subjected to 30 cycles of 1 minute at 96 °C, 1 minute at 55 °C and 30 seconds at 72 °C. The amplified product was hybridized to a set of 43 immobilized oligonucleotides (Table 4), each corresponding to one of the unique spacer DNA sequences within the DR locus. After hybridization, the membrane was washed twice for 10 minutes in 2× SSPE (1× SSPE is 0.18 M NaCl, 10 mM NaOH_2_PO4 and 1 mM EDTA {pH 7.7})-0.5 sodium dodecyl sulfate (SDS) at 60 °C and was incubated in 1:4000-diluted streptavidin-peroxidase conjugate (Boehringer) for 45 to 60 minutes at 42 °C. The membrane was washed twice for 10 minutes in 2 × SSPE-0.5% SDS at 42 °C and rinsed with 2× SSPE for 5 minutes at room temperature. Hybridizing DNA was detected by the enhanced chemiluminescence method (Amersham) and by exposure to x-ray film (Hyper-film ECL; Amersham) as specified by the manufacturer. Patterns were numbered and prefixed with “NH” if from human isolates and “N” if isolated from cattle.

**Table 3:** List of oligonucleotide sequences (primers) and cycling conditions used in the study

Primer name	Primer sequence (oligonucleotide sequences)	Product size	enzyme activation	Cycles [30]			Incubation
				Denaturation	Annealing	Extension	
DRa	GGTTTTGGGTCTGACGAC, 59 biotinylated		96 °C 15 min.	96 °C 1 min	55 °C 1 min	72 °C 30 sec.	42 °C 45–60 min.
DRb	CCGAGAGGGGACGGAAAC		96 °C 15 min.	96 °C 1 min	55 °C 1 min	72 °C 30 sec.	42 °C 45–60 min.

**Table 4:** Sequences of the oligonucleotides used in the study

Spacer No.	Oligonucleotide sequence
1	ATAGAGGGTCGCCGGTTCTGGATCA
2	CCTCATAATTGGGCGACAGCTTTTG
3	CCGTGCTTCCAGTGATCGCCTTCTA
4	ACGTCATACGCCGACCAATCATCAG
5	TTTTCTGACCACTTGTGCGGGATTA
6	CGTCGTCATTTCCGGCTTCAATTTC
7	GAGGAGAGCGAGTACTCGGGGCTGC
8	CGTGAAACCGCCCCCAGCCTCGCCG
9	ACTCGGAATCCCATGTGCTGACAGC
10	TCGACACCCGCTCTAGTTGACTTCC
11	GTGAGCAACGGCGGCGGCAACCTGG
12	ATATCTGCTGCCCGCCCGGGGAGAT
13	GACCATCATTGCCATTCCCTCTCCC
14	GGTGTGATGCGGATGGTCGGCTCGG
15	CTTGAATAACGCGCAGTGAATTTCG
16	CGAGTTCCCGTCAGCGTCGTAAATC
17	GCGCCGGCCCGCGCGGATGACTCCG
18	CATGGACCCGGGCGAGCTGCAGATG
19	TAACTGGCTTGGCGCTGATCCTGGT
20	TTGACCTCGCCAGGAGAGAAGATCA
21	TCGATGTCGATGTCCCAATCGTCGA
22	ACCGCAGACGGCACGATTGAGACAA
23	AGCATCGCTGATGCGGTCCAGCTCG
24	CCGCCTGCTGGGTGAGACGTGCTCG
25	GATCAGCGACCACCGCACCCTGTCA
26	CTTCAGCACCACCATCATCCGGCGC
27	GGATTCGTGATCTCTTCCCGCGGAT
28	TGCCCCGGCGTTTAGCGATCACAAC
29	AAATACAGGCTCCACGACACGACCA
30	GGTTGCCCCGCGCCCTTTTCCAGCC
31	TCAGACAGGTTCGCGTCGATCAAGT
32	GACCAAATAGGTATCGGCGTGTTCA
33	GACATGACGGCGGTGCCGCACTTGA
34	AAGTCACCTCGCCCACACCGTCGAA
35	TCCGTACGCTCGAAACGCTTCCAAC
36	CGAAATCCAGCACCACATCCGCAGC
37	CGCGAACTCGTCCACAGTCCCCCTT
38	CGTGGATGGCGGATGCGTTGTGCGC
39	GACGATGGCCAGTAAATCGGCGTGG
40	CGCCATCTGTGCCTCATACAGGTCC
41	GGAGCTTTCCGGCTTCTATCAGGTA
42	ATGGTGGGACATGGACGAGCGCGAC
43	CGCAGAATCGCACCGGGTGCGGGAG

Source: Kamerbeek et al. [23]

The websites, http://www.pasteur-guadeloupe.fr:8081/SITVIT_ONLINE/ and www.mbovis.org were used in the identification of spoligotypes. Spoligotypes were assigned to families and subfamilies by using the online tools.

#### Data analysis

Data was analyzed using STATA B and Statistical Package for the Social Sciences (SPSS) version 16.0. The 95% confidence interval was calculated online via “the confidence interval of a proportion VassarStats-vassarstats.net/prorp1.html.

## Results

One hundred and sixty slaughtered animals had tuberculous lesions; 128 (80.8%) were emaciated, 18 (11.5%) were slightly emaciated and 14 (7.7%) were looking apparently healthy (Table 5). Sixty-nine (51.9%) out of the 160 infected cattle had lesions in the lung, lymph node and one or more other organs infected (a score of 3+) as predilection site, 49 (32.7%) cattle had predilection sites of lungs and lymph node (2+) and 42 (15.4%) had predilection sites of lungs only based on gross lesions (Table 5).

**Table 5:** Prevalence of bovine tuberculosis in relation to source, breed, age, sex, body conformation and score

Variables	Categories	N (Positive for Culture)	Total	Prevalence (%)	X^2^	*P* Value
Sources	Abadam	1	2	50.0	14.298	0.282
	Bama	3	13	23.1		
	Chad	2	3	66.7		
	Cameroun	0	1	0.0		
	Dalagajeri	1	1	100		
	Damasak	0	2	0.0		
	Damboa	38	107	35.5		
	Dikwa	0	2	0.0		
	Gom	0	2	0.0		
	Gomboru/Ngala	1	4	25.0		
	Gubio	6	12	50.0		
	Gudumbali	0	1	0.0		
	Karenuwa	0	6	0.0		
	Mainok	0	4	0.0		
Total		52	160	32.5		
Breed	Ambala	2	3	66.7	3.525	0.318
	Bokoloji	0	2	0.0		
	Kuri	0	2	0.0		
	Red Bororo	50	153	32.7		
Total		52	160	32.5		
Age	1–5	36	121	29.8	1.709	0.191
	5 and above	16	39	41.0		
Total		52	160	32.5		
Sex	Female	49	154	31.8	0.87	0.351
	Male	3	6	50.0		
Total		52	160	32.5		
Body Confirmation	AH	4	14	28.6	0.11	0.947
	E	42	128	32.8		
	SE	6	18	33.3		
Total		52	160	32.5		
Score	1^+^	8	42	19.0	4.95	0.084
	2^+^	17	49	34.7		
	3^+^	27	69	39.1		
Total		52	160	32.5		

*AH* Apparently Healthy, *E* Emaciated, *SE* Slightly Emaciated, Score (1^+^ = only the lungs affected, 2^+^ = lung and lymphnode infected, 3^+^ = Lung, lymphnode and any other organ infected).

*P*-Value < 0.05 considered significant

One hundred and twenty-one out of 160 cattle (69.2%) between the ages of 1 to 5 had tuberculous lesions, the remaining were above 5 years of age (Table 5). Out of the 160 cattle with tuberculous lesions, 153 (96.2%) were Red Bororo (Rahaji \Abore) and other mixed breed classified as Rahaji due to their similarities, 2 each for the breeds of Bokoloji and Kuri and 3 Ambala (Arab, Bahr el or Ghazal) (Table 5). Majority of the cattle were brought from Damboa local government area (73.1%), other areas include Banki in Bama local government area (LGA) of Borno State, a town sharing border with Cameroun, Gurosaye (border town in Cameroun), Chad Republic, Karenuwa, Mainok, Gamboru/Ngala, Damasak, Abadam, Dikwa (Mafa LGA), Dalajeri and Gudumbali (Table 5). All the categories that were analyzed based on cattle source, breed, age, sex, body conformation and score had *P*-values that were not significant (*P* > 0.05).

**Fig. 4:**
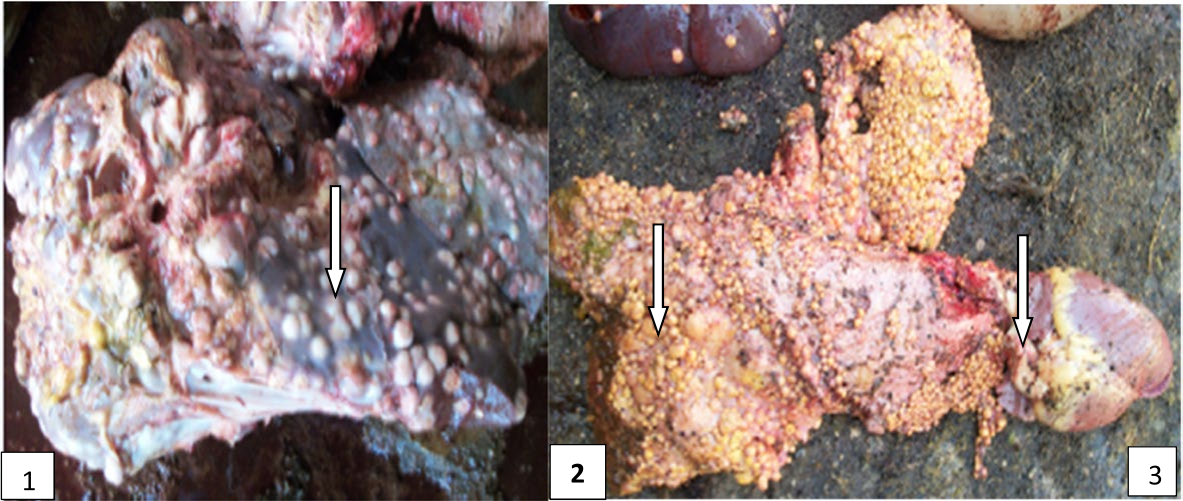
Tuberculous lesions on liver (1), lungs (2) and heart (3)

**Fig. 5:**
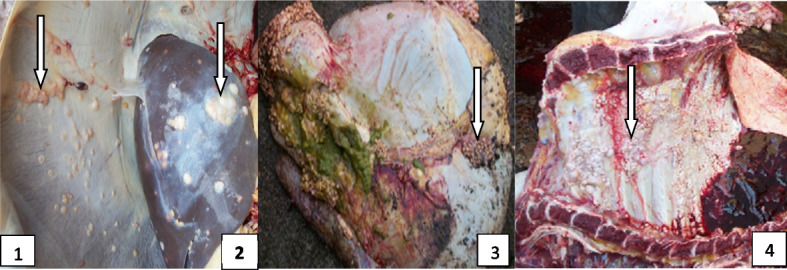
Tuberculous lesions indicated by white arrows on the diaphragm (1), liver (2), stomach (3) and chest cavity (4) observed in Maiduguri abattoir

Out of two hundred and ninety-four (294) tissues that were cultured, 63 tissue samples (from 52 cattle) were culture positive, giving a prevalence of 32.5% (52/160) with 31.8% (49/154) prevalence in females and 50% (3/6) prevalence in males (Figs. 3, 4 and 5 & Table 5). On age ranges, 69.2% (36/52) were between the ages of 1–5 while the remaining 16 (30.8%) were > 6 years. Going by the number of culture positive cattle, 41 (78.9%) were emaciated (E), 6 (11.5%) were slightly emaciated (SE) and 5 (9.6%) were looking apparently healthy (AH). Looking at the predilection sites of infection, 8 (15.4%) out of 52 had a score of 1+ that is only the lung was affected, 18 (32.7%) had a score of 2+ (lungs and lymph node affected) and 26 (51.9%) had a score of 3+ (lungs, lymph node and any other organ affected) (Figs. 3, 4 and 5 & Table 5).

Table 6 shows that lesions from the lungs account for 35.3% of the total grossly infected organs and tissues whereas 98.6% of infected cattle had tuberculous lung lesions, followed by the lymph nodes (27.6%) while 76.9% of the cattle slaughtered had their lymph nodes infected. The list site of infection were the spleen and diaphragm (1.4%) of infected organs and 3.8% affected catted (Table 6).

**Table 6:** Indicating analysis of tissues with tuberculous lesions

S/NO	Organs/parts affected	frequency	Percentage (%) of infected organs	Percentage (%) based on the No of cattle with lesions (160)
1.	Lungs	157	35.3	98.6
2.	Heart	25	5.6	15.6
3.	Spleen	6	1.4	3.8
4.	Liver	20	4.5	12.5
5.	Kidney	9	2.0	5.6
6.	Lymph nodes	123	27.6	76.9
7.	Chest cavity	56	12.6	35.0
8.	Intestine	25	5.6	15.6
9.	Stomach	18	4.0	11.25
10.	Diaphragm	6	1.4	3.8
Total		445	100.0	

A total of 147 sputum samples were analysed; 19/147(12.9%) of the suspected cases were ZN positive while (18/147)12.2% were culture positive. The prevalence of 23.8% (35/147) was obtained from both culture and ZN positive patients. Only 2 of the patients were both ZN and culture positive. For those patients that were HIV positive 6 out of 21(28.6%) of them were either ZN or culture positive. Patients with age ranges from 16 to 30 and 31–45 are worst hit by the disease having a percentage of 77.1% of those that were infected (Table 7). As the age’s progresses, the number of people infected regresses (Table 7). When the positive cases were analyzed, 22 were males (62.9%) and 13 were females (37.1% (Table 7). There was no significant *p*-value (> 0.05) when results for Z-N stain and culture were compared based on sex and age groups (Tables 8 and 9). Analysis from culture positive sputum between abattoir workers and patients from hospitals/clinic indicates a significant difference (*P* = 0.030889) in the prevalence of culture positivity between the two groups of samples (Table 10).

**Table 7:** Positive TB cases based on age category

S/NO	Age ranges	Frequency of +ve cases (Z-N + Culture)	Percentage (%)	Sex	
				Male	Female
1.	1–15	1	2.9	1	–
2.	16–30	14	40.0	7	7
3.	31–45	13	37.1	10	3
4.	46–60	5	14.3	2	3
5.	61–75	2	5.7	2	–
Total		35	100.0	22(62.9%)	13(37.1%)

+ve (positive cases), Z-N (Zielh-Nelseen)

**Table 8:** Ziehl-Neelsen Test results of patients from hospitals/clinic

Category	Ziehl-Neelsen		Total	X^2^	*P*-Value
Sex	Positive	Negative			
F	7	63	70	1.016	0.313
M	12	65	77		
Total	19	128	147		
Age Ranges (Years)					
1–15	0	2	2	2.804	0.581
16–30	11	50	61		
31–45	5	41	46		
46–60	2	20	22		
61–75	1	15	16		
Total	19	128	147

**Table 9:** Sputum culture results from hospitals/clinic

Category	Culture		Total	X^2^	*P*-Value
Sex	Positive	Negative			
Females	7	63	70	0.627	0.429
Males	11	66	77		
Total	18	129	147		
Age Ranges (Years)					
1–15	1	1	2	6.88	0.142
16–30	4	57	61		
31–45	8	38	46		
46–60	4	18	22		
61–75	1	15	16		
Total	18	129	147

**Table 10:** Analysis of sputum culture results from hospitals/clinics and the abattoir

Location	Category	Culture		Total	X^2^	*P*-Value
		Positive	Negative			
	Abattoir	3	79	82	4.6591	0.030889
	Hospital	18	129	147		
Total		21	208	229		

**Fig. 6:**
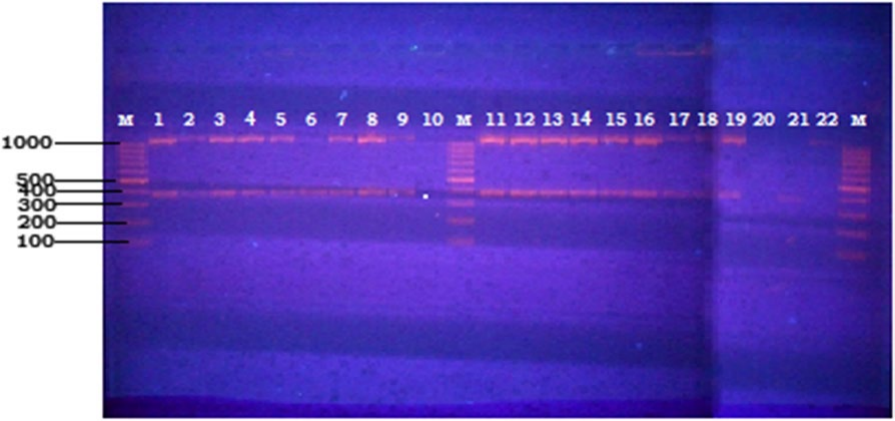
Electrophoretic fractionation of PCR products in 1.5% agar rose from the genus typing of the isolates (obtained from lung lesions), Lanes 1–9 and 11–19: *M. tuberculosis* complex; Lanes 10 & 20: Negative control (Non-template control), Lane 21: H37Rv (Positive control), Lane 22: Non-tuberculous Mycobacteria (NTM), Lane M: molecular weight marker (100 bp) ladder

From the 52 (32.5%) culture-positive growths, 26 (50%) were confirmed belonging to MTC using genus typing (Fig. 6 and Table 11). Deletion typing showed that 17/26 (65.4%) isolates were *M. bovis*. In humans, 229 sputum samples were cultured, abattoir workers [46] and patients (147) from DOTS centers; three isolates from the abattoir and nine from DOTS centers were found to be members of MTC. Of these 12 MTC from humans, seven were characterized as *M. tuberculosis* (58.3%) (Table 5). Spoligotyping of the selected isolates (*n* = 12) revealed SB0944 (*n* = 6) and SB1025 (*n* = 2) in cattle (Table 12) while four spoligotypes SIT 838 and SIT 61 of LAM10_CAM as well as SIT 1054 and SIT 46 of Haarlem families were obtained from humans (Table 12).

**Table 11:** Indicating total number of cattle and humans that were sampled and results of laboratory analysis for culture, Mycobacterium genus typing and deletion analysis

Specie	Total N0. sampled	Culture positive	Mycobacterium Genus typing	Deletion analysis	
			*Mycobacterium tuberculosis* Complex	*M. bovis*	*M. tuberculosis*
Cattle	160	52	26	17	0
Humans	229	21	12	0	7
Total	389	83	38	17	7

**Table 12:**
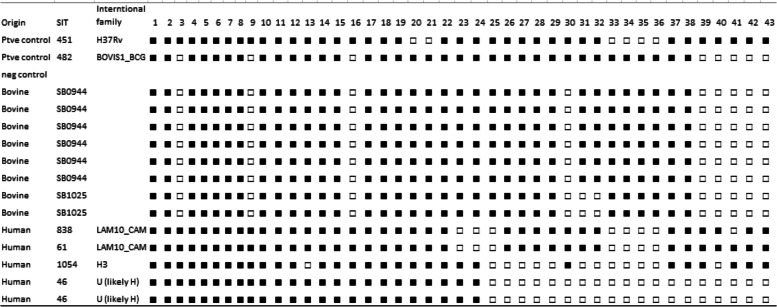
Spoligotypes and the international families of *M. bovis* and *M. tuberculosis* isolated from cattle and humans in Maiduguri

## Discussion

Various breeds of cattle were identified in the course of this study including Ambala which are classified as Zebu breed; Ambala is not a resident breed in the country signifying the movement and cross border trade in Borno state. Kuri breeds identified during this study are classified under Taurines as described by Blench [45]. Majority of cattle with tuberculous lesions were between the ages of 1 to 5 indicating that cattle that are in their active stage of production are more prone to bTB. Majority of cattle slaughtered in Maiduguri abattoir are the Red Bororo breed of cattle.

This study reveals higher prevalence compared to other studies; 9.6% prevalence (9.7% in males and 9.6% in females) obtained by Abubakar et al. [47] in Borno state using ZN staining method; Adedipe et al. [37], using Ziehl Neelsen technique had a prevalence of 5.5 and 8.1% when the samples were cultured from a total of 397 slaughtered cattle were examined at Bodija Municipal Abattoir located in Ibadan, Oyo State; 13.4% prevalence by Nasaka [36] in Uganda where tuberculosis like lesions were collected from cattle and cultured; 6.5% by Ejeh et al. [48] where 248 pathological lesions suggestive of bTB were collected and analyzed using Ziehl-Neelsen microscopy (ZNM); Cadmus et al. [49] had 10.5% prevalence using the simple intradermal test; and Kachalla et al. [50] obtained 17.3% prevalence where serum samples of cattle from Karu and Kubwa abattoirs in Abuja were screened for bTB.

This study has revealed the endemicity of bTB in Maiduguri. The successful culture of mycobacteria from affected tissue demonstrates the presence of generalized or miliary TB in slaughtered cattle in Maiduguri, Borno State. It further demonstrates that all lesions were not detected during routine post-mortem meat inspection at the abattoirs for bTB in carcasses which agrees with the findings of Ejeh [51]. The prevalence rate in males and females revealed that males are more prone to the disease than females based on the result of our culture although the values obtained were not statistically significant. This is contrary to what was obtained in terms of bTB prevalence based on the studies of Ejeh et al. [48] where cows 4.8 and 1.6% for bulls; Opara et al. [52], reported cows (4.5%) than bulls, 3.3%; Cadmus et al. [9], 9.9% cows and 0.6% bulls; Ejeh et al. [53], 7.4% in bulls and 9.9% in cows. Also, the study of Kachalla et al. [50], a prevalence of 29.2% in females and 13.1% in males; Adedipe [37], revealed a prevalence of 6.7% in males and 8.3% in females.

Our finding relates that emaciation is one of the major signs in the identification of tuberculous cattle although obtained values were not statistically significant. Adult cattle with poor body conditions scores were more affected than the young adult with good body condition score. This tallies with the study of Ejeh et al. [48] were a significant association was found between bTB prevalence and body conformation of the cattle that were screened. Likewise, the study of Kachalla et al. [50], also indicated that body condition score is one of the measures in identifying bTB positive cattle, in the study, 50% of the seropositive cattle had poor body condition scores, 17.6% had a fair body condition score and 16.2% had good body condition score. This observation is similar to the report from Adisababa, Ethiopia by Elias et al.*,* [54], which indicated that as body condition scores improved from poor to medium and then to good, the likelihood of positive results significantly decreased. In addition, this result is in agreement with the findings of Ewnetu et al. [55], which reported higher bTB prevalence in cattle with medium than good body condition scores (BCS). Overall, the present result is consistent with previous reports which indicated that animals with good BCS have relatively strong immunological response to the infectious agent than animals with medium BCS and the result could also indicate the wasting nature of the disease [56]. This study also indicates that the greater proportion of the animals had a generalized form of the disease (bTB) based on identified lesions.

In Nigeria, there is inadequate meat inspection in over 90% of our abattoirs [2] and the act of trimming grossly affected parts and passing the other parts as were also observed in the Maiduguri abattoir which exposes the public and most especially those individuals that are immunocompromised such as HIV/AIDS patients to the danger of consuming infected meat. In some of the meat stands infected organs are sold to the public. Inside the abattoir building, those carcasses with generalized tuberculosis (grossly) are trimmed and washed using sponge by the butchers and taken to the stand for sale. This act also contaminates other carcasses and organs that are kept on the floor of the abattoir although Adu-bobi et al. [57] suggested that total rejection cannot be implemented fully when the abattoir management is not in the position to bear the cost of a condemned carcass.

Lungs were most affected followed by the lymph node, chest cavity, the heart and intestine. This further signifies that TB is primarily a disease of the lungs and lymph node before being disseminated to other parts of the body. Also, 12.5% of the affected cattle had lesions in their liver, 15.6% that of intestine and 76.9% of them had infected lymph nodes. This study does not tally with the studies of Opara et al. [52], where lesions were also found in 8.1% of the livers (21.5%), intestines (11.3%) and lymph nodes (11.3%) of the infected cattle.

The study revealed that patients with age ranges from 16 to 30 and 31–45 were worst hit by the disease indicating that over 70% of those that were infected fall between the age ranges of 16–45 years which is in agreement with the findings of Kwaghe et al. [58]. Also, the study of Dim and Dim [59], relates similar findings stating that “the modal age group for new TB positive cases in Enugu state was 25–34 years (29.8%), followed by 35–44 years (20.0%) while 0–14 years contributed the least (1.4%). As the age’s progresses, the number of people infected regresses, 46–60 years and 61–75 years where as only a child was infected between 1 and 15 years of age which agrees with the findings of Kwaghe et al. [58].

There was 3.7% prevalence from the abattoir workers which differs from the findings of Adesokan et al. [27] with 10% (7/70) prevalence and indicated the evidence of exposure to *Mycobacterium bovis* as an occupational hazard to livestock workers. A significant *P*-value was obtained when hospital and sputum samples were compared indicating that there is more likelihood to obtain a TB positive person from the suspected TB positive patients that come for test in the hospitals/clinic. Our study revealed the prevalence of ZN stain method as 12.9% and for culture, 12.2% with an overall prevalence (culture and ZN stain) of 23.8%. The prevalence of our study based on ZN stain only is lower than the overall prevalence that was obtained by Brisibe et al. [60], 23.8% in a decade in Maiduguri (2003–2012) and that of Kida et al. [61] with 26.5% prevalence of sputum smear positivity among patients presenting to the DOTS clinic in Maiduguri but tallies with our overall prevalence.

The isolation of 65.4% *M. bovis* from slaughtered cattle in the study area corroborates earlier findings that indicated *M. bovis* as the primary agent of bTB in cattle. However, the prevalence reported in this study is lower than that of Ejeh [62] who identified 90% of the 40 isolates obtained from organs cultured as *M. bovis*. It is also lower than 99% *M. bovis* out of 180 isolates reported by other authors [26]. Spoligotyping of the selected *M. bovis* isolates revealed the predominance of SB0944 in cattle in the study area. Spoligotypes SB0944 was also detected in camels a study in the same study area [63]. This spoligotype pattern has been previously reported in cattle from Nigeria [29, 64], Chad, Cameroun [65], Mali [66], Morocco [67], France [68] and the United States [69]. Again, the spoligotype SB1025 isolated in this study has been previously reported in Nigeria [26]. As suggested [70], this spoligotype pattern could be generated from SB0944 through a single-step deletion of spacers [26] showing that spoligotypes evolve by the deletion of spacer units only. This further reiterates that SB0944 may represent the spoligotype pattern of the ancestral strain [26]. Importantly, spoligotype patterns SB0944 and SB1025 belong to the African 1 (Af1) clonal complex characterized by the absence of spacer 30 [71] which is also known to be widely distributed in West Africa. Considering the zoonotic nature of *M. bovis*, spoligotype SB0944 had been previously isolated from infected sedentary and trade cattle in Ibadan [64] and from livestock traders at Akinyele Cattle Market in Ibadan [27]. This indicates potential exposure of abattoir workers given prevailing factors that could enhance transmissions such as drinking of unpasteurized milk, processing infected carcasses with bare hands and unguarded close interactions with infected cattle.

Notably, the study also reported *M. tuberculosis* strains belonging to spoligotypes SIT 838 and 61 both of the international family LAM10_CAM. Earlier reports have identified LAM10_CAM in humans in Nigeria [28, 64, 72–74] and other countries in Africa including Cameroon [75], Burkina Faso [76], Sierra Leone [77], Niger and Ivory Coast and parts of Europe [68]. Previous reports showed the LAM10-CAM family as the most predominant circulating clade in Nigeria [28, 72–74, 78, 79] The LAM10-CAM was first described in Cameroon, where it represented 34% of the *M. tuberculosis* isolates in 2003 [75] and has recently emerged as a dominant strain in the western province of Cameroon. Importantly, a study demonstrated LAM 10 as part of spoligotype families including LAM 1 and Beijing families which had the highest sensitivities when compared with isolates belonging to other spoligotype families; suggesting their highly clonal and homogeneous nature [80].

This study revealed two isolates with spoligotypes SIT 1054 and SIT 46 belonging to the Haarlem family. Other studies also reported this spoligotype family within and outside Nigeria [28, 80, 81]. The Haarlem family is considered to belong to modern strains which are known to demonstrate more virulent phenotypes compared to the ancient ones such as the East African and Indian [82]. Further, reports show that the Haarlem family, of European origin, comprises nearly a quarter of the *M. tuberculosis* population in Europe, and that it also accounts for a similar proportion of strains in the Caribbean and Central America [46, 83]. The Haarlem family in these regions is believed to represent a remnant of the post-Columbian European colonization [83, 84]. The Haarlem strains have been associated with multidrug resistance (MDR)-TB population, indicating its ability to cause outbreaks of MDR-TB, following reports from Argentina [85], the Czech Republic [86] and Tunisia [87]. The association between drug resistance (DR) and the Haarlem family were observed in other studies including MDR-TB cases in Tehran, Iran [88], and DR-TB cases in Hungary [89]; where the rates of infection by the Haarlem genotype were 33.5 and 66.2%, respectively [89, 90].

Limitations of the study were; the abattoir workers were not randomly sampled, sampling was done purposively based on the participants’ verbal consent after explaining to them the relevance of the study. Also, sputum samples collected from hospitals/clinics were based on those patients that were likely to be TB positive. This method of sampling may not be generalized, however, the sole purpose of the study was to have an insight on the spoligotypes in the area and the best population for such study is for those participants that were at high risk of being infected with the disease or those that were already indicating the clinical sign of infection. Few isolates were available for spoligotyping due to limited funds. Also, more detailed insights would have been provided if all the isolates obtained were spoligotyped. Furthermore, characterization using such molecular techniques as Mycobacterial Interspersed Repetitive Units-Variable Number Tandem Repeats was not done, as this would have given better epidemiological insights into the circulating strains. It is now common knowledge that spoligotyping has limitations as a tool for the prediction of the exact phylogenetic relationships between strains of the MTC, particularly among modern strains mainly due to homoplasy [46].

Despite these limitations, however, the study reveals SB0944 and SB1025 as the circulating *M. bovis* strains in cattle and LAM 10 and Haarlem families as the circulating *M. tuberculosis* strains among humans in Maiduguri, Borno State, Nigeria.

## Conclusions

This study reiterates that bTB IS endemic in Maiduguri with 32.5% prevalence. Majority of the infected cattle were from Damboa as demonstrated by studies of Igbokwe et al. [91]. This region of the state needs serious attention in order to control bTB in the state. Tuberculosis is also endemic in humans in the study area with a prevalence of 3.7% obtained from the abattoir workers and 12.2% from suspected TB patients from hospitals/clinic. The study concurs with majority of the studies relating TB to people in their active stage of life. The age ranges of 16–30 and 31–45 constitute the majority of those infected (77.1%). The study revealed SB0944 and SB1025 as the circulating *M. bovis* strains in cattle and LAM 10 and Haarlem families as the circulating *M. tuberculosis* strains among humans in Maiduguri, Borno State, Nigeria. We advocate for more extensive epidemiological studies to provide more in-depth insights into the circulating strains MTC among cattle and humans in Nigeria. One Health approach should be in cooperated in dealing with the issue of human and bovine tuberculosis.

## Supplementary Information

**Additional file 1: Supplementary Table 1. **Spoligotype Binary, Spoligotype octal, Spoligo International Type and International family

**Additional file 2: Supplementary Fig. 1.** Electrophoretic fractionation of PCR products in 1.5% agar rose from the genus typing of the isolates (obtained from lung lesions), Lanes 1–9 and 11–19: *M. tuberculosis* complex; Lanes 10 & 20: Negative control (Non-template control), Lane 21: H37Rv (Positive control), Lane 22: Non-tuberculous Mycobacteria (NTM), Lane M: molecular weight marker (100 bp) ladder

## Data Availability

All data generated or analyzed during this study are included in the article.

## Abbreviations

bTB: Bovine tuberculosis
DOTS: Directly Observed Therapy Shortcourse
HIV: Human immunodeficiency virus
MTC: *Mycobacterium tuberculosis* complex
PCR: Polymerase Chain Reaction
TB: Tuberculosis
UMTH: University of Maiduguri Teaching Hospital
