# Supplementary material for: Prevalence and molecular characterization of Mycobacterium tuberculosis complex in cattle and humans, Maiduguri, Borno state, Nigeria: a cross-sectional study
Source: BMC Microbiol. 2023 Jan 9;23:7. doi: 10.1186/s12866-022-02710-y (PMC9827019; doi:10.1186/s12866-022-02710-y)

Table S1: Spoligotype Binary, Spoligotype octal, Spoligo International Type and International family


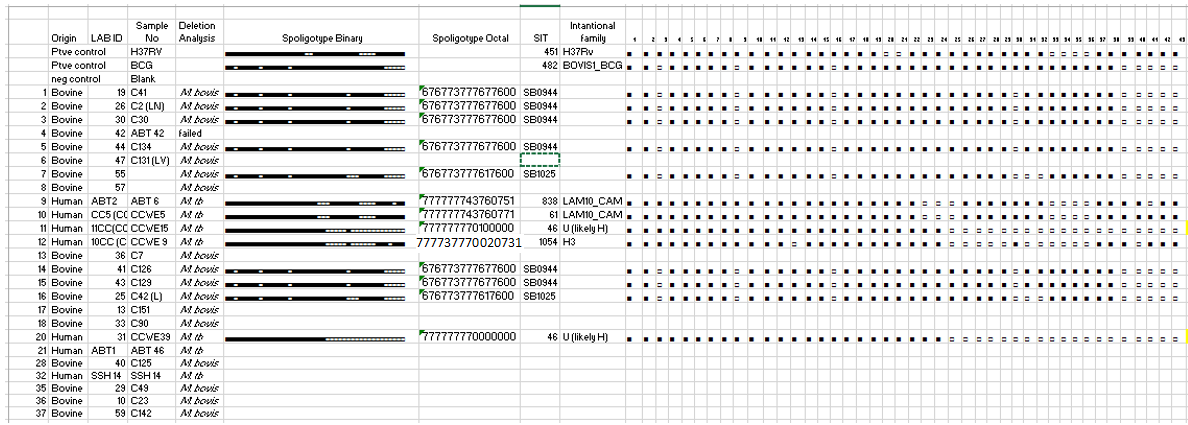

Supplement: Supplementary file 1 — Additional file 1: Supplementary Table 1. Spoligotype Binary, Spoligotype octal, Spoligo International Type and International family [file 12866_2022_2710_MOESM1_ESM.docx]
