# Supplementary figures and images for: Prevalence and molecular characterization of Mycobacterium tuberculosis complex in cattle and humans, Maiduguri, Borno state, Nigeria: a cross-sectional study
Source: BMC Microbiol. 2023 Jan 9;23:7. doi: 10.1186/s12866-022-02710-y (PMC9827019; doi:10.1186/s12866-022-02710-y)

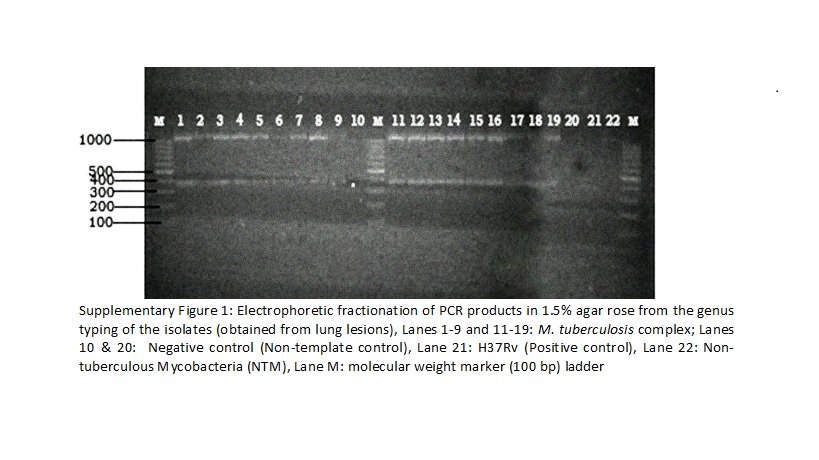

Supplement: Supplementary file 2 — Additional file 2: Supplementary Fig. 1. Electrophoretic fractionation of PCR products in 1.5% agar rose from the genus typing of the isolates (obtained from lung lesions), Lanes 1–9 and 11–19: M. tuberculosis complex; Lanes 10 & 20: Negative control (Non-template control), Lane 21: H37Rv (Positive control), Lane 22: Non-tuberculous Mycobacteria (NTM), Lane M: molecular weight marker (100 bp) ladder [file 12866_2022_2710_MOESM2_ESM.jpg]
